# Effects of home visits by home nurses to elderly people with health problems: design of a randomised clinical trial in the Netherlands [ISRCTN92017183]

**DOI:** 10.1186/1472-6963-4-35

**Published:** 2004-12-15

**Authors:** Ans Nicolaides-Bouman, Erik van Rossum, Gertrudis IJM Kempen, Paul Knipschild

**Affiliations:** 1 Dept. of Epidemiology, Faculty of Health Sciences, Maastricht University, P.O. Box 616, 6200 MD Maastricht, The Netherlands; 2 Dept. of Health Care Studies, Medical Sociology, Faculty of Health Sciences, Maastricht University, P.O. Box 616, 6200 MD Maastricht, The Netherlands; 3 Dept. of General Practice, Faculty of Medicine, Maastricht University, P.O. Box 616, 6200 MD Maastricht, The Netherlands

## Abstract

**Background:**

Preventive home visits to elderly people by public health nurses aim to maintain or improve the functional status of elderly and reduce the use of institutional care services. A number of trials that investigated the effects of home visits show positive results, but others do not. The outcomes can depend on differences in characteristics of the intervention programme, but also on the selection of the target population. A risk group approach seems promising, but further evidence is needed. We decided to carry out a study to investigate the effects in a population of elderly with (perceived) poor health rather than the general population. Also, we test whether nurses who are qualified at a lower professional level (home nurses instead of public health nurses) are able to obtain convincing effects. The results of this study will contribute to the discussion on effective public health strategies for the aged.

**Methods/design:**

The study is carried out as a parallel group randomised trial. To screen eligible participants, we sent a postal questionnaire to 4901 elderly people (70–84 years) living at home in a town in the south of the Netherlands. After applying inclusion criteria (e.g., self-reported poor health status) and exclusion criteria (e.g., those who already receive home nursing care), we selected 330 participants. They entered the randomisation procedure; 160 were allocated to the intervention group and 170 to the control group. The intervention consists of (at least) 8 systematic home visits over an 18 months period. Experienced home nurses from the local home care organisation carry out the visits. The control group receives usual care. Effects on health status are measured by means of postal questionnaires after 12 months, 18 months (the end of the intervention period) and after 24 months (the end of 6-months follow-up), and face-to-face interviews after 18 months. Data on mortality and service use are continuously registered during 24 months. A cost-benefit analysis is included.

The design and setting of the study, the selection of eligible participants and the study interventions are described in this article. Other included items are: the primary and secondary outcome measures, the statistical analysis and the economic evaluation.

## Background

The number of elderly people is increasing. Due to the ageing population, more demands are made on health care services [1]. In the last two decades, preventive programmes have been developed aiming at reducing health care cost and improving the independent functioning of elderly people. One of such programmes is home visitation by public health nurses of elderly people living in the community. This aims to maintain or improve the functional abilities and well-being of elderly people and reduce the use of institutional care services. Such programmes for elderly people are part of national policy in several countries, including the UK, Denmark and Australia. However, the results of trials on the effects of home visits have been inconsistent [2]. Investigators are still in search of the most effective strategy.

In the past years 3 reviews were published on the effects of preventive home visits to elderly people living in the community [2-4]. These used different methodological approaches. Apart from a number of similar trials, each review also included a series of different trials, depending on the inclusion criteria and the date of publication. Van Haastregt *et al *[2] reviewed 15 studies and concluded that no clear evidence exists for the effectiveness of the visits: the observed effects are considered to be fairly modest and inconsistent. Nine trials reported at least one (significant) favourable effect and 6 trials reported no effects. In most of the studies the intervention was aimed at the general population aged 65 years or over, without any selection. The other 2 reviews [3,4] included a meta-analysis of the data and were more positive about the effects of the home visits. Stuck *et al *[4] indicated that home visits can reduce the risk of functional decline and nursing home admission, provided that the interventions are based on a multi-dimensional geriatric assessment and include multiple follow-up visits. Home visiting programmes improved functional status more in people with the lowest mortality risk (younger population, < 80 years). Elkan *et al *[3] reported a favourable effect on mortality and nursing home admissions among members of the general population and frail older people who are at risk of adverse outcomes. However, they did not find improvement in functional status.

One can argue about the differences in the approach of each review, but in general the results of the home visiting studies are heterogeneous with respect to the different outcome measures. Many factors can play a role in the effectiveness of the interventions, including the target population, characteristics of the intervention, the persons carrying out the visits and the compliance to the given advice.

Research in the Netherlands showed that preventive home visits do not seem to be useful for the general population of elderly people [5]. In that trial, experienced public health nurses visited the intervention group (n = 300) at least four times a year over a period of 3 years. The control group (n = 300) received usual care. After 3 years, no or hardly any effects were demonstrated on the health and service use of the total group of visited elderly (see table 1). However, a subgroup analysis indicated that the visits seemed to be effective for elderly with a poor (perceived) health status. Visited persons with poor health at baseline scored considerably better on several health measures (e.g., functional status) compared to similar persons in the control group. Mortality rates after three years were lower (24% versus 40%) and substantial effects were found for referrals to outpatient clinics (61% versus 79%) and also for hospital admissions, especially re-admissions. In the intervention subgroup 47% were admitted at least once to the hospital, with a total of 1,134 days; in the control subgroup these figures were 74% and 2,043 days (table 1). These effects emerged already during the first year of the intervention period [6].

The probable usefulness of home visits for a high risk group was confirmed in five controlled studies [7-11]. However, the results of three other trials did not support this assumption [12-14]. Although home visits for a restricted population seem a promising approach, further evidence is needed. The findings of the earlier Dutch subgroup analysis were based on a relatively small number of subjects (53 in the control and 57 persons in the intervention group). Therefore, we decided to carry out a new trial in which the risk group approach is tested in a larger population of those with (perceived) poor health. At the same time, we appointed nurses who are qualified at a lower professional level (enrolled home nurses instead of public health nurses) to carry out the visits. An experienced public health nurse will supervise them.

This study will investigate the effects of systematic home visits by home nurses to elderly people with (perceived) health problems in terms of their health status, the use of care services and the cost-effectiveness. We expect that the visits will improve the functional abilities, perceived health and quality of life of the participants. We also hypothesize that they will reduce specialist care, institutionalisation, especially hospital (re-) admissions, and total health care expenditures. Evidence regarding the usefulness of the proposed risk group approach is needed to decide on the future implementation of the visits. This article presents the design of this new trial.

## Methods/design

### Study design and setting

**Figure 1:**
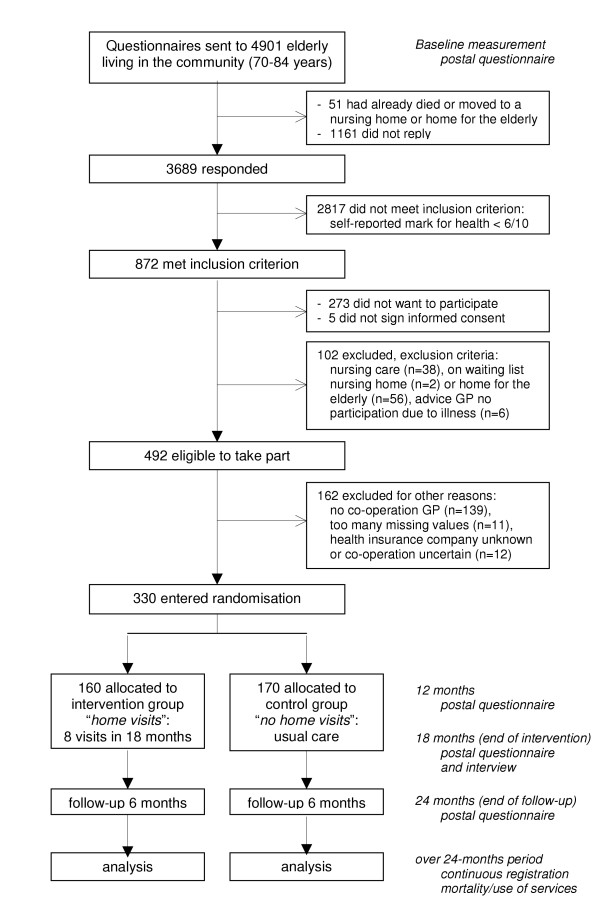
**Study design **The summary of the trial design includes: the screening procedure, the randomisation, the intervention and the points in time at which the effects of the intervention are measured.

The study is carried out as a parallel group randomised trial. It is conducted in co-operation with a large home care organisation in the south of the Netherlands (Sittard and surroundings). The addresses we used to screen eligible persons for the study were drawn from the population register of the municipality. After the screening procedure we randomised 330 elderly. Effects of the intervention are measured by means of postal questionnaires after 12 months, 18 months (at the end of the intervention period) and after 24 months (at the end of a 6-months follow-up period) and by face-to-face interviews after 18 months. Mortality and data on the use of care services are continuously registered over the 24-months research period. A cost-benefit analysis is also included. The design of the study is shown in figure 1. The design is, unless otherwise mentioned, carried out according to plan.

The study has obtained the approval by the Medical Ethical Committee of Maastricht University/Academic Hospital Maastricht.

### Identification of eligible participants

We sent a postal questionnaire to 4901 elderly people between the age of 70 and 84 years who were still living at home. These lived in 14 districts in the research area. We included districts with close proximity to the centre of town where the home care organisation is situated. In this way we limited the travelling time of the nurses to carry out the visits. Districts with large industrial areas were excluded.

Reminders were sent after 2–3 weeks to 45% of the elderly. The response rate was 76% after about 6 weeks. The response rates per district fluctuated between 65% and 81%. The average time to fill out the questionnaire was about 30 minutes. The elderly could do this by themselves or, if they needed help, with the assistance of family, friends or volunteers. A list of names and addresses of volunteers was added to the questionnaire. Even if persons did not want to participate in the study, we kindly requested them to fill out the questionnaire and return it to us. A postage free envelope was included.

The questionnaire was used as a screening instrument and also served as a baseline measurement for the participants of the trial. Among the respondents (n = 3,689, see figure 1), we found 872 persons who reported their health status as poor (on a scale ranging from 1–10 points, report marks 1–5 are considered poor, 6–7 fairly good, and 8–10 good). Our previous home visitation study indicated positive effects for this subgroup. Five persons did not sign the informed consent form and 273 persons with a poor health status did not want to participate in the study. Of the remaining 594 persons, we excluded those who already received home nursing care at baseline, in order to avoid contamination of (other) nursing care. Referral to nursing services after the start of the intervention period has no consequences for the scheduled home visits in the intervention group. It is regarded as a possible effect of the intervention and it is registered as outcome in terms of service use. Persons on a waiting list for admission to nursing homes or homes for the elderly were also excluded. The local independent committee dealing with applications for the use of care services already granted them this service. It is likely that most of them already receive regular supervision of professional caregivers. Six persons were excluded on the advice of their GPs. They were severely or terminally ill and would probably die within 6 months. On the basis of these exclusion criteria, a total of 102 persons were excluded.

After applying the in- and exclusion criteria, 492 persons were eligible to take part in the study. However, we excluded 162 more persons for the following reasons: their GP did not want to co-operate with the study (n = 139), respondents had too many missing values on the functional status scale (n = 11), the health insurance company was unknown (n = 1) or it was uncertain whether the health insurance company would be willing to co-operate (n = 11). The health insurance companies of 96% of the finally selected participants had already given consent to provide us with data on health service use. As we selected persons whose GP was willing to co-operate, relevant health care data from the GP practices are available for all participants. A flow diagram of the selection of participants is shown in figure 1. Finally, 330 persons entered the randomisation procedure. In consideration of the available working hours of the nurses, the maximum number of participants to receive home visits was 160. The control group was hence set at 170.

#### Sample size consideration

We calculated the sample size from the data of our previous home visitation study in the Netherlands [5]. Participants were categorized on the primary outcome measure self-rated health, perceiving their health status as (a) better or the same compared to the start of the study, or (b) worse or deceased. We expect to demonstrate a difference of 20% between the study groups (65% score (a) in group I versus 45% in group II). Based on a 0.9 power to detect a significant difference (α = 0.05, one-sided), 104 participants are required for both study groups. Accounting for a loss to follow up of 30%, we planned to enrol 150 participants per group. This number is also large enough (again extrapolated from our own data) to detect differences in specialist care and institutionalisation (e.g., to detect a difference in mean hospital days of 10 days over a 1.5 year period).

Based on data of the selection criteria we estimated that about 10% of the screened population was eligible for the study (including informed consent). Therefore, we needed to mail a minimum of 3,500 questionnaires to persons aged 70–84 years living in the community. To account for unforeseen circumstances, we decided to send out about 5,000 questionnaires. After applying the inclusion and exclusion criteria, and taking into account the GPs' willingness to co-operate with the study, there were sufficient participants eligible for the study to raise the selected number to 330. This slightly increased the power of the study.

#### Randomisation

The baseline measurements included questions on relevant prognostic factors related to the health status and service use. Before randomisation we divided the 330 participants into two groups: couples (n = 46) and those for whom this did not apply (n = 284). In this way we made sure that eligible persons who lived together, were always allocated to the same study group (in order to avoid contamination of the intervention). The 23 couples were divided into 3 strata on the basis of their (added) score on functional status (0–4, 5–7 or more than 7 out of 18 activities that cannot be carried out independently). The other 284 participants were stratified into 8 strata based on 3 prognostic factors – two health-variables and one service use-variable:

1. functional status (0–2 or more than 2 activities that the elderly cannot carry out independently)

2. changes in health during the 3 months prior to completion of the questionnaire (same/better or worse)

3. contact with a medical specialist in the 3 months prior to completion of the questionnaire (contact yes or no).

The participants in each of the 8 strata were then randomised into either a control or intervention group using a computer generated randomisation list with a block length of 4 [15]. Randomly, we allocated 160 persons to the intervention group and 170 persons to the control group. Table 2 shows the baseline characteristics of the intervention and control group. The study groups are well matched; there were hardly any differences between the groups at the start of the study.

The participants in the intervention group were assigned to one of the three home nurses. This depended on the location of their GP practice, as each nurse was assigned to a number of GP practices. We assumed that this would facilitate the co-operation with the GPs. Each nurse was responsible for 52–56 elderly during the intervention period.

### Study interventions

Our previous study showed that positive effects of the visits for people with a poor health status emerged already within 1.5 years. In the new trial the intervention period is restricted to this period. At the same time we increased the frequency of the visits. This enables the nurses to intervene more promptly on identified problems and risks, and to establish a position of trust in a shorter time period. Experienced home nurses therefore visit the intervention group at least 8 times over an 18 months period. If necessary, extra visits can be made. The duration of the visits can last between 60 and 90 minutes. Participants in the control group receive usual care. As before, they can use or apply for all available services in the area.

Three nurses work half time for the trial. An experienced public health nurse supervises the visits on a weekly basis. All 3 home nurses are recruited from the co-operating home care organisation. Home nurses, as well as public health nurses, are well trained to conduct home visits. They are embedded in community care organisations that traditionally have preventive tasks. The home nurses are not part of a multidisciplinary team, but advice can be obtained from in-home specialists within the home care organisation, e.g., a dietician, a diabetes specialist and an occupational therapist. A nurse geriatric specialist from the local hospital can also be consulted, if necessary. At regular intervals, once every 6–8 weeks during the intervention period, he also advises the nurses on important geriatric issues.

#### Home visit protocol

The home visits can be described as "systematic home visits to elderly people with health problems carried out by a home nurse". The 3 most important elements of the visits are (1) to detect problems or risks, (2) to give advice and (3) to refer to other professional or community services. This brief description is applicable to all home visiting studies that have been carried out so far. However, there are large differences in the protocols that have been used in earlier studies, ranging from an interview to collect information on social and health conditions [16] to a 'multidimensional geriatric assessment' in which medical, functional, psychosocial, and environmental evaluation of the problems and resources are assessed [12,17]. Earlier studies did not show any clear relation between the structure of the visits and the effects. So far, the active components of the intervention are not known yet, but a number of elements seems to be of importance for the contents of the visits. We tried as much as possible to include these elements into the protocol: e.g., face-to-face assessment, good communication between the nurse and the elderly including an empathic attitude by the nurse, an individual plan, a client-centred approach, good compliance with the given advice and multiple visits [4,18].

The visits are carried out in a systematic way according to a nursing model [19] that distinguishes 4 steps: diagnosis, planning of activities, carrying out the activities and evaluation.

##### Diagnosis

Our starting-point is a client-centred approach. The elderly can indicate which problems they experience and which needs they have. The EasyCare Questionnaire [20,21], an elderly assessment system, is used to detect further problems. Also, additional checklists are used on a variety of topics: e.g., vision, hearing and use of medication. A number of instruments are used for further diagnostic assessment: the get-up-and-go test [22], the Geriatric Depression Scale [23] and the Mini Mental State Examination [24]. During the visits no physical examination takes place, as the home nurses are not qualified to do so. If necessary, the elderly are referred to their GPs.

##### Planning of activities

An individual plan for each elderly person is set up. The activities are planned in agreement with the elderly, as this will improve compliance. We only included elderly with a poor (perceived) health, hence a broad range of problems can come forward, including physical, mental as well as social problems. Guidelines on a number of geriatric topics are used for advice and referral regarding problems and risks that are identified. A Handbook of Nursing Diagnosis [25] is also used to set up goals and interventions. A maximum of three problems (and 2 interventions per problem) is being dealt with at one visit. Among the planned activities are referrals to professional or community services, and advice or information is given regarding, e.g., nutrition, social and physical activities and home aids.

##### Carrying out the activities

The elderly are primarily themselves responsible to carry out the planned activities. The home nurse only supports the elderly. In order to improve compliance, the nurses contact the elderly by telephone 1 to 4 weeks after each visit, depending on the type of advice. They ask whether the advice has been followed, and if not, what the impediments are and if further assistance is necessary. The participants are offered consultation with the nurses by telephone each morning between 9.00 – 9.30 hours.

##### Evaluation

The evaluation of each home visit takes place at the next visit. The cycle is then repeated and new or old, but not solved, problems can be dealt with.

In the 3-months period before the start of the visits, the home nurses were actively involved in the development of the visiting protocol. They also received relevant training in communication skills and using assessment tools. They took courses on several subjects, e.g., relevant geriatric health topics, behaviour change and the usage of the Handbook of Nursing Diagnosis [25]. Several pilot visits were carried out, in which different aspects of the protocol were trained, e.g., using assessment tools and measuring instruments.

Communication between the nurses and the GPs is according to the 'normal' communication lines between nurses of the home care organisations and the GPs. Before the start of the study all GPs received a list of eligible participants registered at their practice, to screen very ill persons. After randomisation a definite list of participants was sent to them, but no reference was made to which treatment group they belong. The allocation of the participants to the 2 groups was disclosed after conclusion of the first 3 home visits. The GPs then received an overview of all treated problems for each participant in the intervention group, including the accompanying recommendations and the results of the interventions. The GPs were asked for their comments or suggestions and in this way they could become involved, if they wanted to. A similar overview will be sent to them for visits 4–6 and 7–8.

#### Process evaluation

All elements of the intervention are monitored as part of a process evaluation. This includes the registration of topics discussed at each visit, treated problems, advice given and referral to other services. The evaluation of each visit is registered at each next visit and includes the compliance with the given advice. Reasons for non-compliance are noted. The nurses' experiences with the visiting protocol, the role of the supervising public health nurse and the patient's experiences with the home visits will be assessed at the end of the intervention period by means of face-to-face-interviews.

Other aspects of the intervention process assessed are: the time spent on the visits, including the travelling and preparation time and the time spent on telephone contacts. Elements of the telephone conversation after each visit, most importantly whether the elderly complied with the given advice, are registered.

Detailed analyses of the intervention process and outcome data might help to identify which programme characteristics are related to possible favourable effects of the visits and may result in the development of more effective interventions. It might also provide additional information for the possible implementation of the visits in daily practice.

#### Outcome measures

The primary health related outcome measures are: self-rated health, functional status, quality of life and changes in self-reported problems. In addition, a variety of other health measures (secondary outcome measures) will be assessed. Information will be obtained, among other things, on health complaints, medication use, and loneliness and mental health. The municipality will supply mortality data (secondary outcome measure) over the entire research period.

The use of services relates to the frequency and duration of care from the following services: domestic and community nursing care, GP, physiotherapy, day care in institutional care settings, hospital outpatient clinics, hospital, nursing home, home for the elderly, use of aids and modifications to the home. The primary outcomes for service use are specialist medical care and hospital (re-) admission. The health insurance companies will supply data on the use of services over the two-year research period. Additional data not covered by the health insurance companies, will be supplied by GPs, the hospital, the home care organisations, etc. Table 3 shows an overview of the outcome measures, their operationalisation and at which time points the measures are carried out.

### Statistical analyses

The main analyses will be conducted according to the intention-to-treat principle. Analysis of primary and secondary endpoints will be performed using relevant significance tests (e.g., chi-square, t-test or analysis of variance). Regression techniques will be used, if necessary, to estimate the effects for the various outcome measures, adjusting for small differences between the groups at the start of the study. In addition, we will conduct per-protocol analyses; these are restricted to those participants who complied fully with the intervention protocol and outcome measurements. Preplanned subgroup analysis will be performed for the following subgroups: living alone/together; health deterioration over the previous 3 months; functional status and locus of control. Differences in approaches between the nurses will be investigated.

#### Economic evaluation

A cost-effectiveness analysis will be carried out in which we consider costs from a societal perspective. The economic evaluation will measure and evaluate the 'real' costs. In this study we will include direct health care costs, i.e. costs made for the home visit programme and health care costs made by the participants. Costs of the intervention programme consist of costs for the screening procedure, salaries of the nurses, travel expenses, costs of training sessions for the nurses, etc. Health care costs include costs of inpatient and outpatient treatment, consultation by GPs and other medical practising specialists, physiotherapy, medication, professional home care, nursing home, meals on wheels, aids and appliances, etc. In order to estimate the costs, the quantity of each resource will be multiplied by its assigned unit cost of price.

Direct non health care costs (e.g., the travel costs made by participants) are not included. These should preferably be gathered prospectively by means of a cost-diary [26]. We considered this too burdensome for the participants. Indirect health care costs (costs which are made during extra gained years of life) and indirect non health care costs (the value of production lost to society due to illness-related absence from work and days of inactivity) are often also included in an economic evaluation. We decided however not to include those costs, because of their limited relevance in a population of retired elderly people.

#### Time plan for this study

The screening procedure was carried out in the fall of 2002. In January 2003 we sent a letter to the elderly notifying them that they were selected to participate in the study and whether they would receive home visits or not. In February the home visits started. They are carried out according to plan and will end in September 2004. The first effect evaluation, 12 months after the start of the intervention period, has taken place: 302 questionnaires were sent out in March 2004. The response rate was 95%. Since the beginning of the intervention period, a total of 24 participants died and 4 persons withdrew from the study. Three more effect evaluations will take place: two evaluations after the intervention period in October 2004 (a postal questionnaire and a face-to-face interview) and one after the 6 months follow-up in March 2005 (postal questionnaire).

## Discussion

The use of postal questionnaires turned out to be a good and inexpensive method to screen elderly people – there were more than sufficient eligible persons to participate in the research project. The response rate was high and less than one percent of the questionnaires were omitted due to too many missing values. For most of the variables, the percentage of missing values varied between 0 and 2 per cent. Media coverage shortly before sending out the questionnaires and accompanying letters from the municipality and the university may have contributed to the high response rate. It is not certain whether the results are comparable to other (larger) towns in the Netherlands. The response rate of the postal questionnaires used for the first effect evaluation (12 months after the start of the study) was 95%. Nearly all included elderly seemed to be motivated to participate.

We selected elderly with a poor perceived health status, because we expected the home visits to be more beneficial for this group rather than for those who are still in good health [5]. Results from the data analysis of the first postal questionnaire (the screening instrument and the baseline measurement) showed that the eligible persons indeed scored worse on most health related variables, including functional status, mental health and social functioning [27].

We considered including a third group of elderly with poor health status to receive home visits from voluntary workers. This was, however, not feasible, mainly because the number of participants with a participating GP was too low. The frequency of the visits and the level of professionalism, nurses versus voluntary aids (usually without any professional qualifications), could be a topic of study in another trial depending on the outcome of this study.

## Competing interests

The author(s) declare that they have no competing interests.

## Authors' contributions

Erik van Rossum and Paul Knipschild were responsible for the research question. They contributed to drafting of the study protocol, as did Ruud Kempen. Ans Nicolaides-Bouman made the first draft of this paper. The other 3 authors commented on it and approved the final version.

## Pre-publication history

The pre-publication history for this paper can be accessed here:



## Figures and Tables

**Table 1:** Mortality and use of services (percentages) for all participants and for those who rated their health status as poor at the start of the study

**Results of study by van Rossum *et al *[5]**	**Total population**		**Population with poor perceived health**	
**4 home visits a year over 3 years in intervention group; no visits for the control group (usual care)**	Intervention group (n = 292)	Control group (n = 288)	Intervention group (n = 57)	Control group (n = 53)

**Mortality**	41 (14%)	49 (17%)	14 (24%)	21 (40%)
**Referrals to outpatient clinics**	132 (55%)	166 (66%)	27 (61%)	38 (79%)
**Hospital (re-) admissions ** **number of days**	121 (41%) 3,838	133 (46%) 4,789	27 (47%) 1,134	39 (74%) 2,043
**Admission to home for the elderly**	20 (7%)	18 (6%)	12 (21%)	7 (13%)

**Table 2:** Baseline characteristics of study participants

**Characteristic**		**Intervention group n = 160**	**Control group n = 170**
**Age**		75.8 (3.7)	75.6 (3.9)
**Gender**			
male		64 (40%)	68 (40%)
female		96 (60%)	102 (60%)
**Composition of household**			
alone		53 (34%)	61 (36%)
together		103 (66%)	108 (64%)
**Education**			
primary school		60 (39%)	65 (39%)
lower/middle professional education		81 (52%)	92 (55%)
higher professional education		15 (10%)	11 (6%)
Self-rated health*			
1–4		62 (39%)	67 (39%)
5		98 (61%)	103 (61%)
Functional status**			
Adl-dependencies	0	73 (46%)	81 (48%)
	1–11	86 (54%)	89 (52%)
Iadl-dependencies	0–1	76 (49%)	83 (50%)
	2–7	79 (51%)	82 (50%)
Total number of dependencies	0–2	83 (53%)	92 (55%)
	3–18	75 (47%)	76 (45%)
**Health change in previous 3 months**			
same/better		85 (53%)	82 (48%)
worse		75 (47%)	88 (52%)
**Health affects social participation**			
often		82 (53%)	87 (51%)
sometimes		49 (32%)	50 (29%)
never		24 (16%)	33 (19%)
**Contact GP in last 3 months**			
yes (no is remaining %)		140 (88%)	150 (88%)
**Contact specialist in last 3 months**			
yes (no is remaining %)		108 (69%)	117 (70%)
**Hospital admission in last 3 months**			
yes (no is remaining %)		21 (13%)	24 (14%)
**Use of home care**			
yes (no is remaining %)		64 (40%)	61 (37%)

* Indicated by a report mark on a scale ranging from 1 to 10 points. Participants with a poor health status were included (report mark below 6).

** Refers to 11 activities of daily living (Adl) and 7 instrumental activities of daily living (Iadl) or housekeeping activities (GARS). Adl / Iadl-dependencies: indicates the number of activities for which the elderly are dependent on others in order to carry out the activity.

**Table 3:** Outcome measures and their operationalisation

**Outcome measure**	Operationalisation*	Measurement**
Self-rated health***	report mark between 1–10	0, 1, 2, 3
Functional status***	GARS [28], score 18–72 Adl, subscore 11–44 Iadl, subscore 7–28	0, 1, 2, 3
Quality of life***	Rand-36 [29], 1item SF-20 [30], 2 subscales, score 0–100	0, 1, 2, 3
Changes in self-reported problems***	3 main problems, 3-points scale	0, 1, 2, 3
**Health complaints**	SCL-90 [31], 2 subscales	4
**Depressive complaints**	GDS [23], score 0–15	4
**Mental status**	MMSE-12 [32], score 0–12	4
**Locus of control**	Mastery Scale [33], score 7–35	4
**Social support**	SSL12-I [34], score 12–48	4
**Loneliness**	Loneliness Scale [35], score 0–11	4
**Medication**	volume, costs	4
**Aids and modifications to the home**	type, costs	4, 5
**Mortality**	number	5
Use of extramural and institutional care, e.g., medical specialist help*** and hospital (re-) admission***	e.g., number of contacts GPs, days in hospital, costs	5

* the underlined scores indicate the most favourable score on the specific scale

** 0 = postal questionnaire at the start of the study

1 = postal questionnaire after 12 months

2 = postal questionnaire after 18 months (at the end of the intervention period)

3 = postal questionnaire after 24 months (at the end of a 6-months follow-up period)

4 = face-to-face interview after 18 months

5 = continuous registration by services over a 24-months period

*** primary outcome measures
